# Supplementary material for: Arabidopsis thaliana Sucrose Phosphate Synthase A2 Affects Carbon Partitioning and Drought Response
Source: Biology (Basel). 2023 May 6;12(5):685. doi: 10.3390/biology12050685 (PMC10215923; doi:10.3390/biology12050685)
Supplement: Supplementary file 1 [file biology-12-00685-s001.zip › biology-2367584-supplementary.pdf]

biology

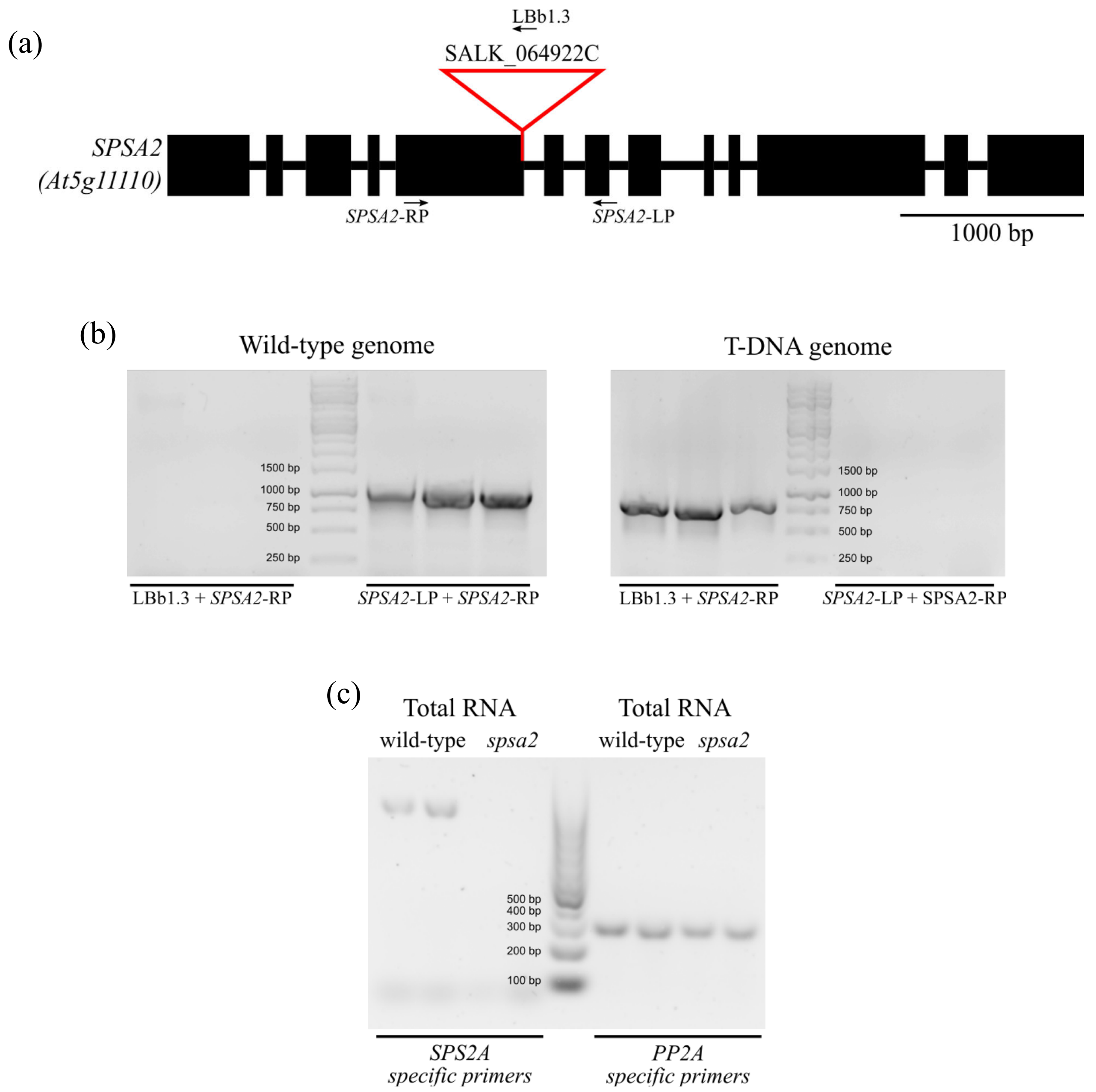

Figure S1. T-DNA insertion in the SALK_064922C homozygous line: **(a)** Gene structure of At5g11110. Primers used to screen the SALK_064922C line, indicated by arrows, were derived from the SALK T_DNA primer design web tool (http://signal.salk.edu/tdnaprimers.2.html). Black bars: exons; black lines: introns. **(b)** Selection of homozygous line through PCR amplification of wild-type allele using *SPSA2*-LP and *SPSA2*-RP primers, and T-DNA band using LBb1.3 and *SPSA2*-RP primers. Left: genomic DNA from wild-type plants; right: genomic DNA from T-DNA line. The two images are taken from the same agarose gel and cropped for illustration purposes. Three plants for each genotype were analyzed. **(c)** Expression level of *SPSA2* in wild-type and T-DNA plants. Total RNA was extracted from two wild-type and two homozygous (*spsa2*) plants and retrotranscribed, and cDNA was used as template for RT-PCR analysis using *SPSA2*- and *PP2A*-specific primers (see Table S1). Samples were separated in a single agarose gel; the image was cut out for illustration purposes.

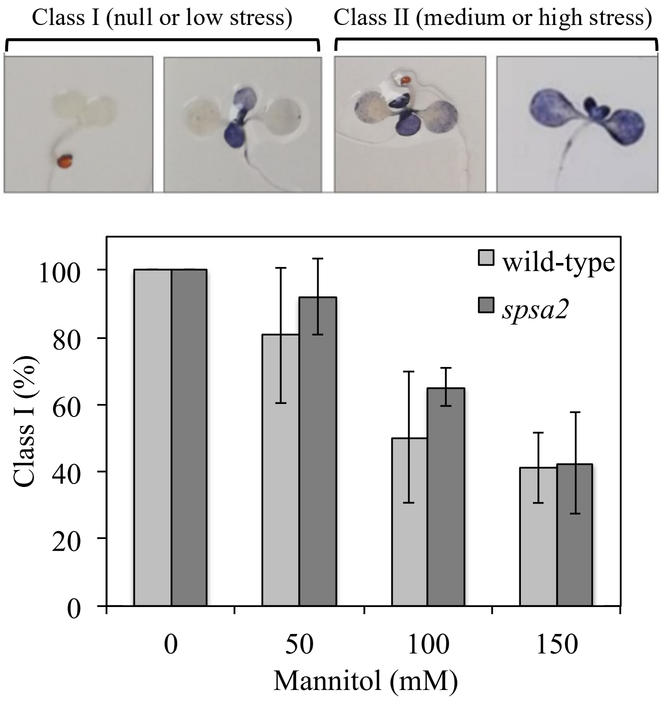

Figure S2. NBT-staining for qualitative evaluation of oxidative stress. Upper panel: color scale used to assess the level of stress. ROS content was measured on 2-week-old wild-type and *spsa2* plants exposed to different concentrations of mannitol. Data are means ± SDs (*n*=30 for each genotype and each condition). A t-test was performed, and no significant statistical differences were observed.

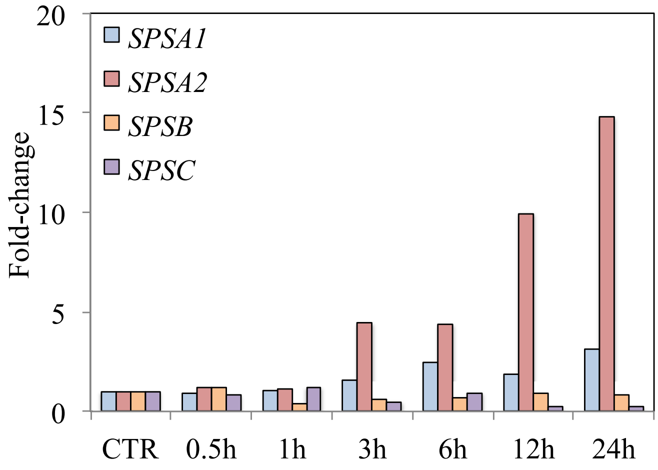

Figure S3. Relative expression levels of the four *SPS* genes in response to drought (300 mM mannitol). Data are from eFP Browser [41].

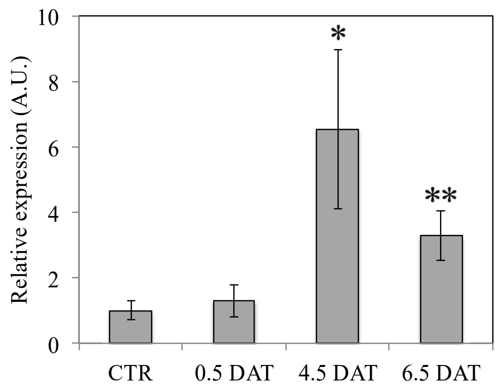

Figure S4. Expression profile of *SPSA2* in response to drought. Expression levels of *SPSA2* in wild-type plants exposed to 150 mM mannitol were analyzed using qPCR. Three independent biological samples were analyzed. Values are reported as means ± SDs. The t-test was used for statistics: * $p < 0.05$; ** $p < 0.01$.

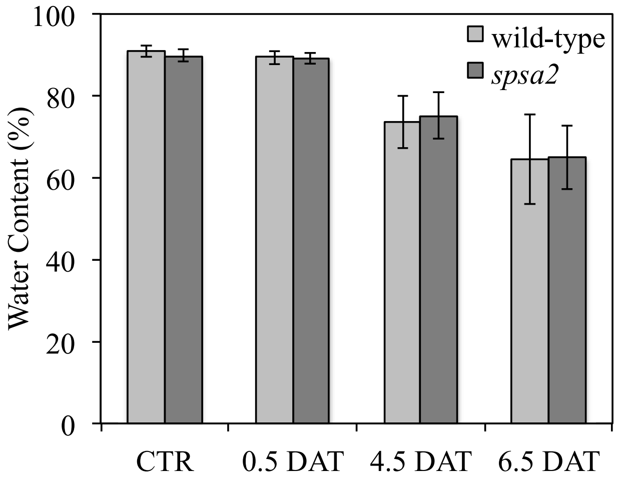

Figure S5. Relative water content in wild-type and *spsa2* plants measured under control and stress conditions. Plants were harvested at 12 h light. A minimum of 20 plants were collected for each genotype and for each experimental point. Values are reported as means ± SDs. A t-test was performed, and no significant statistical differences were observed.

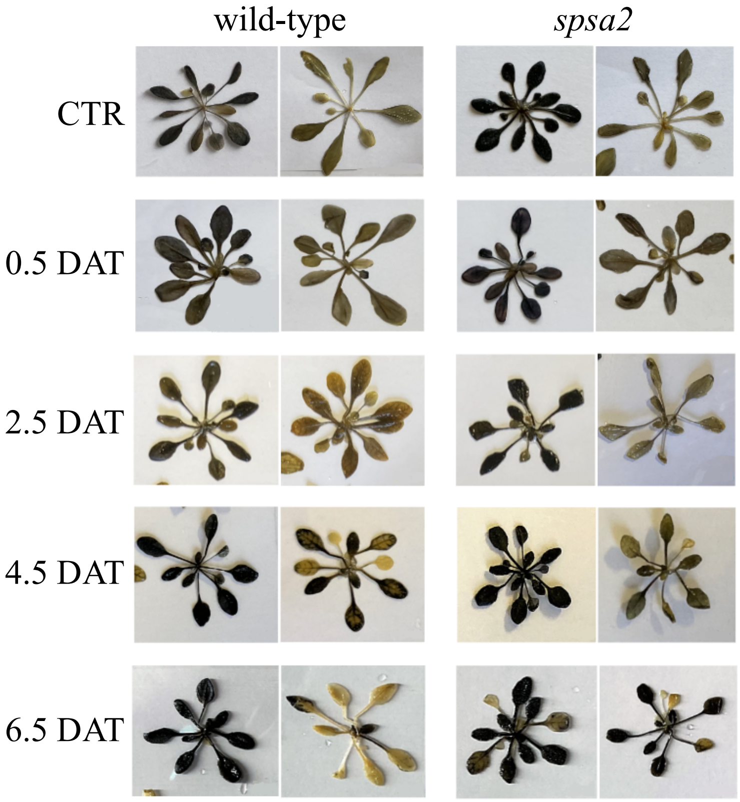

Figure S6. Starch content in wild-type and *spsa2* was evaluated with Lugol staining. Plants were collected at 12 h light. For clarity, two stained plants for each genotype, and experimental data are reported. Plants were chosen to represent the typical range of colors observed.

Table S1. List of primers.

	Left primer	Right primer
LBb1.3		5'-ATTTGCCGATTTCGGAAC-3'
[1]*SPSA2*-LP		5'-CCAGCTACTCTGAACCGTCTG-3'
[1]*SPSA2*-RP	5'-TGCAAGACTTCAAGGTTCGC-3'	
[2]*SPSA2*	5'-GCAAGAGCGAGAATCATAGGCT-3'	5'-CCAGCTACTCTGAACCGTCTG-3'
[3]*SPSA2*	5'-AGTGAAAGATCCCGCTTTGA-3'	5'ACCTAAGGGCCTGAGATCGT-3'
ACT	5'-AACTCTCCCGCTATGTATGTCGC-3'	5'-CAATACCGGTTGTACGACCACTG-3'
PP2A	5'-GTAGGACCGGAGCCAACTA-3'	5'- CATCCTTACCCAAGACTGGA-3'
G6PD1 (At1g09420)	5'-GCAGCATGGGATCTATTCAC-3'	5'-ACCAACAGGACCTCTGCTTC-3'
G6PD2 (At1g24280)	5'-AAGTGACGAGCTTGATGCAG-3'	5'-ACCACGGCTACCATAAGGAT-3'
G6PD3 (At3g27300)	5'-GCTTTTCACTCCATTGCTCA-3'	5'-TAGATAATGGGCACCGACTG-3'
G6PD4 (At5g13110)	5'-TTGCTGAAGAAACTAGAGCTG-3'	5'-TGATGCAATGATGGATCTGAG-3'
G6PD5 (At5g35790)	5' - GACACAATCAGAGGCGACCA - 3'	5'- TAGCTGATCTGCTTCCGCTG - 3'
G6PD6 (At5g40760)	5' - TGGGAGAAAATGACGGAAGC- 3'	5'- TCAACGTGCCATTGACCAGA- 3'

[1]*SPSA2*, pair of primers used in the selection of homozygous *spsa2* plants;
[2]*SPSA2*, pair of primers used in the detection of the residual expression level of *SPSA2* in homozygous line;
[3]*SPSA2*, pair of primers used in the detection of the expression level of *SPSA2* in wild-type plants in response to drought. Primers were as in [15].
